# Is boosting the immune system in sepsis appropriate?

**DOI:** 10.1186/cc13787

**Published:** 2014-03-24

**Authors:** Jean-Marc Cavaillon, Damon Eisen, Djilalli Annane

**Affiliations:** 1 Unit Cytokines & Inflammation, Institut Pasteur, 28 rue Dr. Roux, 75015 Paris, France; 2 Victorian Infectious Diseases Service, Royal Melbourne Hospital, 300 Grattan Street, Parkville 3050 Victoria, Australia; 3 Department of Medicine, Royal Melbourne Hospital, University of Melbourne, Victorian Infectious Diseases Service, Royal Melbourne Hospital, 300 Grattan Street, Parkville 3050 Victoria, Australia; 4 Intensive Care Unit, Hôpital Raymond Poincaré, 104, boulevard Raymond-Poincaré, 92380 Garches, France

## Abstract

A relative immunosuppression is observed in patients after sepsis, trauma, burns, or any severe insults. It is currently proposed that selected patients will benefit from treatment aimed at boosting their immune systems. However, the host immune response needs to be considered in context with pathogen-type, timing, and mainly tissue specificity. Indeed, the immune status of leukocytes is not universally decreased and their activated status in tissues contributes to organ failure. Accordingly, any new immune-stimulatory therapeutic intervention should take into consideration potentially deleterious effects in some situations.

## Introduction

Refinements of supportive care of patients with severe sepsis have decreased their overall mortality, but no adjuvant drug therapy has emerged despite strenuous efforts in the field. Twenty years have passed since the first patients with sepsis were included in clinical trials based on the understanding that TNF orchestrates the inflammatory response and should be the target for therapeutic intervention. In response to the failure of therapies aiming to target either the up-stream microbial activators or the effector molecules of the inflammatory cascade, a new concept has emerged of boosting the immune system to counter immunosuppression that develops in patients who survive the initial, hyperinflammatory period of sepsis [1].

Inflammation is a highly sophisticated and complex response that fundamentally ‘aims’ to protect the host. In this review, we argue against the promulgation of what we believe is a misleading perception of sepsis inducing secondary immunosuppression. The possible negative consequences of immune system-boosting therapy are so great that we believe such an approach should be considered with great caution.

It is important to realize that events occurring in patients with sepsis are not a simple dichotomy resulting from a balance between pro-inflammatory and anti-inflammatory mediators. Oversimplification may have led to previous therapeutic failures. Maintaining such an over-simplistic analysis of inflammation may lead to further failed trials, compounding our current state of therapeutic futility in sepsis. By reviewing the immunological events of sepsis, we also attempt to understand why we have already spent over 20 fruitless years trying to reverse it with targeted adjuvant therapies.

## Arbitrarily classifying cytokines as pro- or anti-inflammatory mediators is unreliable

Classifying cytokines as pro- or anti-inflammatory has led to oversimplification of the inflammatory response. Consequentially, especially in a disorder as complex as sepsis, simplistic therapeutic approaches have been considered. The inappropriateness of this classification is widely illustrated by studies revealing some paradoxical behaviors of so-called pro- and anti-inflammatory cytokines. In regard to TNF, the prototypic ‘pro-inflammatory’ cytokine, TNF-treated murine macrophages produced less IL-12 and IL-23 after IFNγ and lipopolysaccharide (LPS) stimulation [2]. *In vivo*, TNF induces extra-adrenal production of immunoregulatory glucocorticoids in the intestinal mucosa during acute intestinal inflammation [3]. Similar observations have been reported for other pro-inflammatory cytokines such as IL-1 and IFNγ. In a dextran sulphate sodium-induced colitis model, mice deficient in IL-1RI signaling showed increased susceptibility to and failed to mount a protective type I interferon response after Toll-like receptor (TLR) 9 ligand administration [4]. Furthermore, IFNγ knockout (KO) mice had significantly greater endotoxin-induced uveitis as compared with wild-type mice, and the injection of murine IFNγ suppressed the severity of endotoxin-induced uveitis in both wild-type and KO mice [5].

Paradoxical properties have also been reported for IL-10, the prototypic anti-inflammatory cytokine. Its pro-inflammatory activity been established in human volunteers receiving endotoxin injection [6]. Our own *in vitro* studies showed that adherence of human monocytes modulated the effect of IL-10 on expression of 16 genes, including ‘suppressor of cytokine stimulation’ (SOCS) molecules, in the opposite direction as compared with non-adherent cells [7]. These observations illustrate the statement by Moore and colleagues that ‘IL-10 can effect very different outcomes depending on timing, dose, and location of expression. In some scenarios, the expected immuno-suppressive activities are observed, while in others, IL-10 enhances immune or inflammatory responses’ [8].

Among other cytokines classified as anti-inflammatory, transforming growth factor-beta (TGFβ) may behave as pro-inflammatory mediator as TGFβ-transgenic mice are more sensitive to LPS-induced shock [9] and some of its inflammatory activities reflect its capacity to favor the differentiation of T helper (Th)17 and production of the pro-inflammatory IL-17. The classification of non-cytokine inflammatory mediators also relies on an overly simplistic division between pro- and anti-inflammatory properties. This is well illustrated by prostaglandin E_2_ (PGE_2_), a key mediator of infectious immunopathology. On one hand, PGE_2_ induces fever, increases vascular permeability, increases vasodilatation, and causes pain while also inhibiting production of TNF, increasing production of IL-6, inhibiting 5-lipoxygenase and leukotriene A4 generation, and inducing 15-lipoxygenase and the generation of the lipoxins involved in inflammation resolution. On the other hand, PGE_2_ has inhibitory properties on macrophages, neutrophils, Th1 lymphocytes, natural killer (NK) cells, and cytotoxic lymphocytes but activates mast cells, Th2, Th17, and regulatory T lymphocytes (T_reg_) [10]. This panoply of PGE_2_-stimulated events amply demonstrates the inability to simply characterize the activities of this and the other molecules mentioned as pro- or anti-inflammatory.

## Sometimes-ambiguous roles of cytokines in infection and sepsis

In an early anti-TNF monoclonal antibody intervention study, a significant improvement in day 3 survival was observed between the antibody-treated group and the placebo group [11]. Although this was not a pre-specified primary outcome, it is interesting to see that the treatment targeting TNF consisting of a single early injection was beneficial within a short period of time after sepsis onset, reinforcing the idea that TNF plays a key deleterious role in the early events of sepsis. Once anti-TNF treatments were better targeted to the sickest patients by adding biological inclusion parameters (plasma IL-6 level), survival was significantly improved on day 28 [12].

Synergistic effects between immune modulators are a key characteristic of their effect. This explains how a non-lethal dose of one cytokine can lead to mortality when injected with a non-lethal dose of another cytokine. Similarly, it may explain how the removal of some inflammatory mediators by coupled plasma filtration-adsorption was protective in an endotoxin-shock model while levels of circulating bio-active TNF were unaffected [13].

Clear demonstrations of cytokine-mediated tissue damage exist. Nevertheless, because of their ambiguous role mentioned above, identification of their precise role during sepsis has led to controversy. In animal models of sepsis, the role of TNF may vary depending upon the type of infection [14]. Many model parameters influence conclusions of the relative role of the different mediators studied. Identical cytokines have been found to be protective or deleterious depending upon the model. This has been the case for IFNγ [15] and granulocyte-macrophage colony-stimulating factor (GM-CSF) [16] among others (for example, IL-17, IL-33, ‘TNF-related apoptosis-inducing ligand’ (TRAIL), and TGFβ).

## Opposing effects of immune cells in sepsis

Host-protective innate immune responses and consequent inflammation are inextricably linked and overlapping. Consequently, the same cellular actors are key elements defending the host against infection while simultaneously contributing to deleterious events. For example, neutrophil extracellular traps that catch and kill bacteria and fungi are associated with the release of elements such as histones and mitochondria that behave like damage-associated molecular patterns perpetuating the inflammatory process.

Beneficial or deleterious roles of the same leukocyte subset have been reported depending upon the experimental model. For example, a peritonitis model using nude mice (lacking T cells) suggested that T lymphocytes contribute to protective immune responses [17]. By contrast, in an *Escherichia coli* sepsis murine model, T lymphocytes markedly contributed to severity [18]. Similarly, T_reg_ improved survival in polymicrobial sepsis [19] whereas, in another report, reduced T_reg_ activity led to improved survival [20]. The ‘half angel/half devil’ role of NK cells during severe infection is also described. NK cells contribute to systemic inflammation during polymicrobial sepsis but play a critical protective role in host defense against *Staphylococcus aureus* lung infection (as reviewed in [21]). Although apoptosis of dendritic cells (DCs) is particularly increased during sepsis, they are protective in murine polymicrobial sepsis [22]. Transcriptomic analysis of DCs in trauma patients shows a large number of upregulated inflammatory genes, suggesting their contribution to systemic inflammation and organ failure [23].

Apoptosis of lymphocytes, DCs, and NK cells is a hallmark of sepsis. Hotchkiss and colleagues [24] provided key experiments demonstrating that lymphocyte apoptosis was deleterious and its prevention highly protective. In addition to the depletion of apoptotic lymphocytes that contribute to the alteration of the immune status, apoptotic T cells themselves can further produce an immunosuppressive milieu following their release of TGFβ [25]. In contrast, the apoptosis of neutrophils is reduced. Interestingly, injection of apoptotic neutrophils in LPS-challenged mice with or undergoing cecal ligature puncture improved outcomes [26]. This may be due to the capacity of apoptotic neutrophils to limit the production of IL-1 and TNF by LPS-activated monocytes and to favor the production of IL-10 and TGFβ [27]. Favoring neutrophil apoptosis while differentially preventing that of lymphocytes and DCs would represent a considerable interventional challenge!

## Inflammatory mediators and their effects in various organ compartments promote organ failure

**Figure 1:**
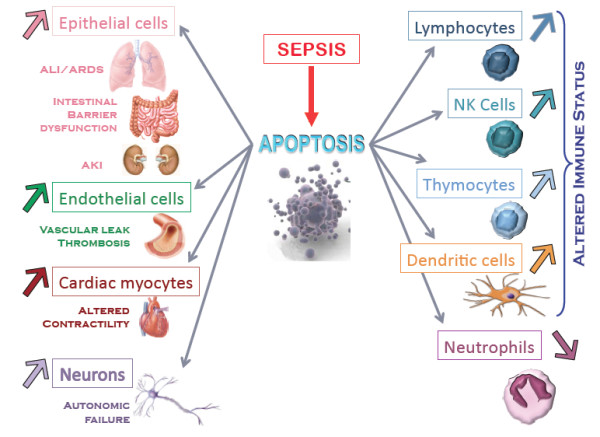
**During sepsis, many types of cells (but not neutrophils) display enhanced apoptosis, leading to various deleterious consequences.** AKI, acute kidney injury; ALI/ARDS, acute lung injury/acute respiratory distress syndrome; NK, natural killer.

In sepsis, apoptosis does not only affect immune cells. Apoptosis of epithelial cells, endothelial cells, neurons, and cardiac myocytes is reported with crucial effects of loss of altered barrier function (Figure 1): in the lungs - acute lung injury and adult respiratory response syndrome [28]; in the kidneys - acute kidney injury [29]. Enhanced translocation of bacteria and bacterial products occurs consequent on intestinal epithelial cell apoptosis [30], contributing to the concept of the gut as the motor of multiple organ failure (MOF). Sepsis-induced cardiac myocyte apoptosis produces altered contractility and cardiac dysfunction [31]. Apoptosis of endothelial cells [32] induces vascular leakage. Finally, microglial and neuronal apoptosis may follow autonomic failure that precedes shock and MOF [33].

In addition to epithelial apoptosis, tight junction alterations enhance organ dysfunction. It has been demonstrated that nitric oxide favors disruption of epithelial cell tight junctions in numerous organs, including liver, gut, and lung. Leukotrienes favor protein extravasation as shown in the kidney of septic mice [34].

Still, cytokines remain the main orchestrators of these tissue injuries. In a model of acute kidney injury, it was nicely demonstrated that inflammatory cytokines, including TNF and IL-17, cause small intestine and liver injury [35]. Among others, IL-17A is critical for generation of intestinal ischemia/reperfusion injury and subsequent liver and kidney injury [36]. All together, the altered functions of epithelial cells, endothelial cells, neurons, and cardiac myocytes contribute to MOF that may influence outcomes in sepsis more than altered immune status.

## The concomitant occurrence of inflammation, anti-infectious response, and altered immune status in sepsis

When Roger Bone coined the concepts of systemic inflammatory response syndrome (SIRS) and compensatory anti-inflammatory response syndrome (CARS), he conceived that one or the other would be predominating [37]. However, we contend that CARS should be considered an adapted compartmentalized response with the aim of silencing some acute pro-inflammatory genes and maintaining the expression of certain genes involved in the anti-infectious process. Despite our views [38], authors still propose a two-wave concept with SIRS appearing before CARS, although they admit that ‘rigorous examination of previous studies provides evidence that both pro-inflammatory and opposing anti-inflammatory response(s) occur concomitantly in sepsis’ [1].

Tamayo and colleagues [39] studied a large panel of circulating cytokines in patients with SIRS or sepsis, concluding that both pro- and anti-inflammatory mediators play roles from the very beginning of this life-threatening condition. Similarly, meta-analysis of 12 transcriptomic studies including 784 individuals led to the conclusion that ‘the arbitrary distinction of separating sepsis into pro-inflammatory and anti-inflammatory phases is not supported by gene-expression data’ [40].

Immune status has been studied frequently by measuring TNF or other inflammatory cytokine production by circulating monocytes in response to LPS [41]. We studied patients undergoing abdominal aortic surgery, showing that reduced expression of human leukocyte antigen (HLA)-DR on CD14^HIGH^ monocytes occurs during surgery [42]. Similarly, HLA-DR expression was already reduced on monocytes taken very soon after severe trauma at accident scenes [43]. Altered TNF production capacity of circulating cells in response to TLR2 or TLR4 agonists is also observed very soon after injurious insults, such as on admission of patients after cardiac arrest [44]. Even if soon after the initial insult the intensity of the inflammatory response reaches its peak, there is a persistent inflammation associated with altered immune status in surviving patients [45]. The more severe the insult, the more profound is the alteration and the more chance the patients have to develop adverse clinical outcome.

## The immune status of leukocytes during sepsis and SIRS varies depending on the compartment in which they reside

Terms such as ‘immunoparalysis, immunosuppression, and anergy’ are far too extreme to describe the immune status of circulating leukocytes in patients with sepsis or SIRS. Altered immune status of circulating leukocytes is not globally present. Indeed, some functions like phagocytosis remain unaltered [46], and *ex vivo* cytokine production in response to heat-killed *S. aureus* (HKSA) remains unchanged in patients with sepsis [47] compared with healthy controls. This is in full agreement with the observation that LPS primes HKSA-induced TNF production in macrophage cell lines instead of leading to cross-tolerance [48]. While the concept of endotoxin tolerance is considered to partially mimic the alteration of immune status in sepsis, it is worth mentioning that cross-tolerance between microbial agonists is not invariant. For example, *Candida albicans* and fungal cell wall β-glucan also prime LPS-induced pro-inflammatory cytokine production [49].

These observations led us to propose the concept of leukocyte reprogramming [50] to explain the fact that tolerised macrophages retain anti-infectious properties. In addition, in tissues, there are numerous examples to illustrate the hyper-activity of these cells. For example, in mice with polymicrobial sepsis alone or as a ‘second hit’ after traumatic hemorrhage, it was nicely demonstrated by Chaudry’s group [51] that the *ex vivo* production of TNF or IL-6 after LPS activation was significantly reduced among peripheral blood mononuclear cells and splenic macrophages but that it was enhanced in alveolar and Kupffer cells. Similarly, in a murine model of trauma, the cytokine productive capacity of Kupffer cells and alveolar macrophages was enhanced [52]. Indeed, macrophage functions differ depending on the compartment from which they derive. We established [53] that the specific cytokine and cellular microenvironment within the lung was responsible for this particular resistance of alveolar macrophages to endotoxin tolerance, which can also be observed in human alveolar macrophages [54]. Similarly, in kidneys, in response to a second challenge with LPS, the expression of TNF and inducible nitric oxide synthase was further enhanced [55]. This may explain why unilateral nephrectomy could be protective in a murine peritonitis model and after LPS injection [56]. Most importantly, despite the fashionable concept of M1/M2 macrophages, the response of macrophages to IL-4 and IFNγ is in fact completely different depending upon their origin [57]. As a consequence of this great heterogeneity of immune cells within different compartments, each tissue behaves independently, contributing to the global inflammatory response with a specific pattern, as illustrated by differential cytokine expression in liver, lungs, heart, brain, muscle, kidney, intestine, and spleen [58]. Another example of the different behavior of leukocytes in various compartments is the frequent occurrence of hemophagocytosis (>60%) directly observed in the bone marrow of the critically ill [59]. This phenomenon is associated with extreme production of inflammatory cytokines. Accordingly, it has been proposed that when hemophagocytosis is diagnosed in critical care patients, aggressive immunosuppressive therapy be undertaken without delay [59].

Differences between cells harvested from different compartments after sepsis have also been reported for spleen and peritoneal myeloid DCs [60]. The major differences between compartments are further illustrated by the fact that gene deficiency may differentially affect outcomes of infection. For example, IL-10 deficiency protects against *Francisella tularensis* pulmonary infection but aggravates cutaneous infection [61]. Similarly, we showed that scavenger receptor-A (SR-A), ‘macrophage associated receptor with a collagenous base’ (MARCO), CD36, or TLR2 deficiency protect mice against peritoneal *S. aureus* infection while these deficiencies aggravated pneumonia [62]. Interestingly, when *Streptococcus pneumoniae* was the pathogen used to colonize the murine nasopharynx, MARCO KO mice (but not SR-A KO mice) had significantly impaired clearance of pneumococcal colonization [63].

Furthermore, inflammatory foci cells may not behave similarly to cells from other healthy compartments. For example, it was shown that neutrophils derived from sputum of patients with chronic bronchitis or cystic fibrosis are insensitive to inhibitory effects of IL-10 in contrast to circulating neutrophils [64].

## Murine models poorly mimic the clinical settings

The concomitant presence of inflammation within tissues and altered immune status within the hematopoietic compartment is short-lived in murine models rendering them inappropriate to study patients with concomitant sepsis and CARS [65]. In addition, mice are highly resistant to bacteria like *S. aureus* and their serum contains factors that limit inflammatory response intensity as compared with human serum [66]. A most provocative report comparing transcriptomic patterns of circulating cells from trauma patients, human endotoxemia-model participants, and murine-model equivalents revealed total absence of correlation [67]. When most therapeutic approaches have been validated in preclinical studies performed with murine models, one understands why those were not the most appropriate ones.

The scientific community needs to reconsider models used to validate therapeutic approaches. If murine responses do not resemble human processes, maybe other species, like the pig, should be preferred. Porcine monocytes and LPS-activated macrophages are closer to their human counterparts than murine cells [68]. Of course, murine models remain valuable to further decipher the mechanisms of sepsis. The best example is the two-hit model, which demonstrated that the nature of the first hit and its severity, the nature of the infection, and the route of infection may influence the outcome in a completely opposite direction [69].

## Are patients with sepsis dying of immune failure - dissecting the arguments used to describe compensatory immunosuppression occurring after sepsis?

The clinical observations used to argue that immunosuppression occurs in sepsis patients surviving the initial inflammatory cascade [1] are in essence that patients develop nosocomial infections due to opportunistic pathogens, including reactivated chronic viral infections, and that patients who die after sepsis have unresolved foci of infection. These underpinning observations require further consideration.

Representing bacteria such as *Enterococcus faecium*, *Stentrophomonas maltophilia*, and *Pseudomonas aeruginosa* along with Candida as ‘opportunistic pathogens’ overstates the role of sepsis-induced immune dysregulation as the primary cause of nosocomial infection in these patients. These multiply-instrumented, high-intensity care, bed-bound, vulnerable patients often have breaches in their integument and mucous membranes (airways, surgical sites, indwelling catheters) and perturbed microbiomes from antibiotic treatments. Overgrowth of antibiotic-resistant microorganisms and barrier defects predispose them to secondary infections, even without overt defects in their immune defenses [70]. These are all organisms of normal virulence that cause nosocomial infections in sepsis patients because of the selection pressure of potent antibiotics and the presence of biofilm affected/colonized intravascular and urinary catheters.

Additionally, reactivation of herpes simplex virus (HSV) and cytomegalovirus (CMV) may have some clinical relevance in critically ill patients. CMV-emia is quite common in patients with sepsis (30% in some studies) and is at least associated with worse outcome in ICU patients in recent meta-analyses. Whether CMV could cause immune compromise itself, be a reflection of immune compromise, or simply be an indicator of poor outcome in patients with sepsis remains unclear [71]. Reactivation of oro-labial HSV is extremely common in sepsis, and HSV can frequently be detected in respiratory secretions. However, only one study has reliably investigated lower respiratory tract infection in critically ill, immunocompetent patients, showing that 21% of patients with ventilator-associated pneumonia (VAP) had bronchopneumonitis due to HSV [72]. In 55% of these patients, the VAP appeared to be due to HSV alone. However, acyclovir treatment had no impact on the outcome in patients with HSV bronchopneumonitis [72]. Of greater relevance to predisposition to nosocomial infection in sepsis patients remaining in ICUs for prolonged periods are physical breaches in innate immune system barriers. Intravascular catheters, endotracheal tubes with consequently increased dead space, and increased gastric pH due to peptic ulcer prophylaxis regimens are all, along with broad-spectrum antibiotics, potent promoters of nosocomial infection.

Post-mortems (PMs) identifying unresolved infection foci are not reliable proof that patients are dying of sepsis. Pneumonia is frequently present in patients in whom supportive care is withdrawn due to failure to thrive. Where pneumonia has been found more frequently at PM than was appreciated ante-mortem, the extent of pulmonary involvement was not quantified [73]. In this series, there was clear agreement by clinical and PM assessment that MOF was the commonest cause of death [73]. These data call into question the relevance of unresolved, PM infection in patients dying in the ICU as a direct indicator of immunosuppression following as a direct consequence of previous sepsis. If the patients die with infectious foci and altered immune status, it does not mean they die because of them.

## Boosting the immune system

Because of the monocyte deactivation in sepsis, it was proposed to restore it with the use of either IFNγ or GM-CSF, two cytokines that counteract endotoxin tolerance. The first attempt was successfully performed in nine septic patients who received subcutaneous IFNγ that restored *ex vivo* cytokine production and HLA-DR expression by monocytes [74]. The authors claimed that overall mortality was lower in the treated group compared with historical controls. In mechanically ventilated trauma patients, IFNγ was aerosolized. However, in a previous phase III study in burn patients, IFNγ had failed to protect patients from infection or decrease mortality [75]. We must recall that IFNγ injection increases mortality in animal models of polymicrobial infection [15]. All together, these data have limited the routine use of IFNγ in ICU patients, although a Dutch clinical trial is ongoing.

GM-CSF has been demonstrated to be able to restore some immune status parameters. However, a meta-analysis concluded that GM-CSF did not significantly reduce in-hospital mortality, although it significantly increased infection recovery [76]. Although no adverse effects were reported, it is worth recalling a case report of a patient who developed a fatal adult respiratory distress syndrome after GM-CSF treatment [77]. In animal models, GM-CSF favors LPS-induced lung inflammation, amplifying LPS-induced bronchoconstriction [78]. GM-CSF favors production of TNF and IL-1. In a recent study, it was confirmed that GM-CSF synergizes with LPS, promoting IL-1β secretion [79]. Lethal injection of LPS in GM-CSF receptor KO mice led to far lower mortality among these mice as compared to wild type mice. Given all the efforts made by some authors to convince the scientific community of the use of GM-CSF, it is challenging to read the conclusion of this present paper given that GM-CSF has been previously underestimated as a target for therapeutic intervention in many bacterial infections and inflammatory disorders associated with the production of IL-1β.

IL-7 is another cytokine that is promoted for the treatment of sepsis and that is supported by murine and human *ex vivo* tissue data [1,80]. One can conjecture that systemic treatment with IL-7 may act in undesired places, as illustrated by the following: IL-7 worsens graft-versus-host-induced tissue inflammation [81]; favors inflammation in colitis [82], contributes to arthritis severity [83]; upregulates chemokines, IFNγ, macrophage recruitment, and lung inflammation [84]; and, finally, increases production of inflammatory cytokines by monocytes and T cells [85].

Many other cytokines (for example, IL-2, IL-12, IL-15, and TNF) can boost the immune system and are reported to be beneficial in murine sepsis models. However, one wonders whether systemic treatment with any immunostimulating cytokine may act on tissue leukocytes boosting the inflammatory process while boosting immune status as well. In this perspective, the attempt to treat peripheral mononuclear cells of sepsis patients *ex vivo* with IL-2 before re-injecting them is an interesting approach that prevents the delivery of this cytokine to the bloodstream, allowing it to act strictly on the desired cells [86]. In this study, the mortality was 8% in the extracorporeally treated group of patients (n = 121) but was 21% in the patients receiving standard treatment (n = 52).

## Approaches for innovative therapeutic interventions

Rather than repeating the mistakes of past experimental treatments for sepsis in which therapies were developed after successful preclinical models that may be far from mimicking human disease, it would be ideal to proceed in the future with new treatments in which extensive human data are available prior to embarking on expensive licensure studies. Furthermore, identifying currently licensed drugs with tolerable safety profiles as potential sepsis agents leap-frogs costly drug development and early-phase human studies.

In animal models, extant licensed drugs, such as chloroquine [87] and androstenenediol [51], have successfully restored immune status. Most interestingly, in the latter case, the treatment protected mice against polymicrobial sepsis and boosted altered *ex vivo* cytokine production observed with peripheral blood cells and spleen macrophages, dampening production observed with alveolar macrophages and Kupffer cells. A similar compartmentalized adapted specificity was reported with estradiol [88].

Other approaches involve pro-resolving lipid mediators [89], although it is uncertain whether they may also adversely boost immune status. The recently recognized aspirin-triggered lipoxins, anti-inflammatory mediators of inflammation resolution, make aspirin a possible inexpensive agent for both prevention and treatment of sepsis. Considerable observational cohort data show improvements in mortality in patients with sepsis pretreated with aspirin [90]. This approach is being prospectively studied as part of an aspirin primary prevention trial.

Could other immunomodulatory approaches be considered with less putative dangerous consequences on inflamed tissues. This may be the case of thymosin-α1. Indeed, a very promising study demonstrated its efficiency to improve clinical outcome in patients with severe sepsis [91], after a preliminary investigation had demonstrated a better performance with respect to organ failure scores in thymosin-α1-treated patients with sepsis arising from intra-abdominal infection due to carbapenem-resistant bacteria [92]. However, one must call for caution since thymosin-α1 can also favor the production of inflammatory cytokines and nitric oxide and further increases the percentage of T_reg_ cells [93,94]. Still, very little is known of its effect on leukocytes present in different compartments.

The cell surface molecules containing in their intracytoplasmic domain an immunoreceptor tyrosine-based inhibition motif - such as programmed death-1 (PD-1), B and T lymphocyte attenuator (BTLA), and cytotoxic T-lymphocyte antigen 4 (CTLA-4) - could also be interesting targets for new therapeutic approaches. The expression of PD-1 on T cells and its ligand (PD-L1) on monocytes is upregulated in critically ill [95] or septic shock [96] patients. Increased expressions were associated with increased occurrence of secondary nosocomial infections and mortality after septic shock [97]. Not only are PD-1-deficient mice markedly protected from the lethality of sepsis, accompanied by a decreased bacterial burden and suppressed inflammatory cytokine response [98], but also blockade of PD-1 or PD-L1 improves survival in a murine model of sepsis, reverses immune dysfunction, inhibits lymphocyte apoptosis, and attenuates organ dysfunction [99-101]. The relevance of these observations in human settings is still needed. CTLA-4 is a high-avidity receptor for CD80 and CD86. Enhanced CTLA-4 expression was demonstrated more frequently in patients with sepsis than in non-infected critically ill patients or control subjects [102], and blocking CTLA-4 improved survival in bacterial and fungal experimental sepsis [103,104]. However, the use of such an approach seems tricky since, in animal models at high dose, anti-CTLA-4 could worsen survival [103], and the use of Abatacept (a soluble CTLA-4 dimerized with an Fc fragment of immunoglobulin) led to increased survival in invasive pneumococcal infection [105]. Similarly, BTLA expression is enhanced in patients with SIRS or sepsis [106] and, in a murine model of sepsis, BTLA-deficient mice displayed an enhanced resistance [107]. In contrast, these mice displayed enhanced susceptibility to endotoxin-induced shock [108]. Accordingly, the exact role of BTLA needs to be further deciphered before strategies targeting BTLA could be proposed to treat patients with sepsis.

## Conclusions

**Figure 2:**
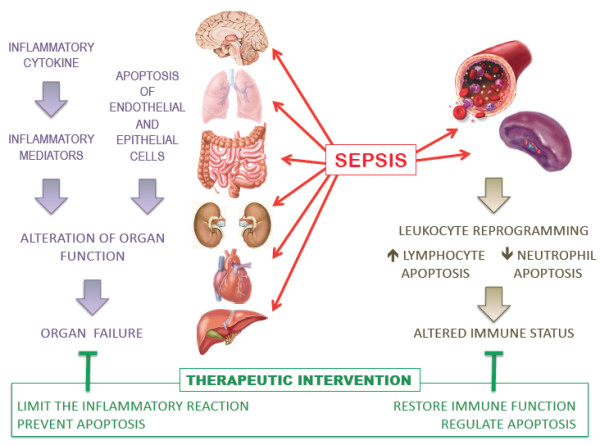
New therapeutic interventions should address both the events in the tissues that lead to organ failure and the altered immune status of leukocytes restricted to some specific compartments.

New therapeutic approaches to treat sepsis should take into consideration that the immune status of leukocytes in the peripheral blood might be quite different from those present in inflamed tissues. We believe that a systemic approach to immune stimulation is not appropriate if immune cells are boosted generally, independent of their location. An ideal drug would limit the overzealous inflammatory process that leads to organ failure and favor homeostatic responsiveness of leukocytes (Figure 2). This is the challenge we have to address if we wish to avoid further decades of disillusionment.

## Abbreviations

BTLA: B and T lymphocyte attenuator; CARS: Compensatory anti-inflammatory response syndrome; CMV: Cytomegalovirus; CTLA-4: Cytotoxic T-lymphocyte antigen 4; DC: Dendritic cell; GM-CSF: Granulocyte-macrophage colony-stimulating factor; HKSA: Heat-killed *Staphylococcus aureus*; HLA: Human leukocyte antigen; HSV: Herpes simplex virus; IFNγ: Interferon-gamma; IL: Interleukin; KO: Knockout; LPS: Lipopolysaccharide; MARCO: Macrophage-associated receptor with a collagenous base; MOF: Multiple organ failure; NK: Natural killer; PD-1: Programmed death-1; PD-L1: Programmed death-1 ligand; PGE2: Prostaglandin E_2_; PM: Post-mortem; SIRS: Systemic inflammatory response syndrome; SR-A: Scavenger receptor-A; TGFβ: Transforming growth factor-beta; Th: T helper; TLR: Toll-like receptor; TNF: Tumor necrosis factor; Treg: Regulatory T lymphocyte; VAP: Ventilator-associated pneumonia.

## Competing interests

The authors declare that they have no competing interests.

## Authors’ contributions

J-MC and DE wrote the review. DA read and amended it. All authors read and approved the final manuscript.

## References

[B1] Hotchkiss RS Monneret G Payen D Immunosuppression in sepsis: a novel understanding of the disorder and a new therapeutic approach Lancet Infect Dis 2013 13 260 268 10.1016/S1473-3099(13)70001-X 23427891 PMC3798159

[B2] Zakharova M Ziegler HK Paradoxical anti-inflammatory actions of TNF-alpha: inhibition of IL-12 and IL-23 via TNF receptor 1 in macrophages and dendritic cells J Immunol 2005 175 5024 5033 16210605 10.4049/jimmunol.175.8.5024

[B3] Noti M Corazza N Mueller C Berger B Brunner T TNF suppresses acute intestinal inflammation by inducing local glucocorticoid synthesis J Exp Med 2010 207 1057 1066 10.1084/jem.20090849 20439544 PMC2867273

[B4] González-Navajas JM Law J Nguyen KP Bhargava M Corr MP Varki N Eckmann L Hoffman HM Lee J Raz E Interleukin 1 receptor signaling regulates DUBA expression and facilitates toll-like receptor 9-driven antiinflammatory cytokine production J Exp Med 2010 207 2799 2807 10.1084/jem.20101326 21115691 PMC3005235

[B5] Fukushima A Ozaki A Ishida W Fukata K Ueno H Systemic interferon-gamma suppresses the development of endotoxin-induced uveitis in mice Curr Eye Res 2005 30 7 12 10.1080/02713680490522461 15875359

[B6] Lauw FN Pajkrt D Hack CE Kurimoto M van Deventer SJH van der Poll T Proinflammatory effects of IL-10 during human endotoxemia J Immunol 2000 165 2783 2789 10946310 10.4049/jimmunol.165.5.2783

[B7] Petit-Bertron AF Pedron T Gross U Coppée JY Sansonetti PJ Cavaillon JM Adib-Conquy M Adherence modifies the regulation of gene expression induced by interleukin-10 Cytokine 2005 29 1 12 15579372 10.1016/j.cyto.2004.09.001

[B8] Moore KW De Waal Malefyt R Coffman RL O’Garra A Interleukin-10 and the interleukin-10 receptor Annu Rev Immunol 2001 19 683 765 10.1146/annurev.immunol.19.1.683 11244051

[B9] Garcia-Lazaro JF Thieringer F Lüth S Czochra P Meyer E Renteria IB Galle PR Lohse AW Herkel J Kanzler S Hepatic over-expression of TGF-beta1 promotes LPS-induced inflammatory cytokine secretion by liver cells and endotoxemic shock Immunol Lett 2005 101 217 222 10.1016/j.imlet.2005.06.003 16054705

[B10] Kalinski P Regulation of immune responses by prostaglandin E2 J Immunol 2012 188 21 28 10.4049/jimmunol.1101029 22187483 PMC3249979

[B11] Abraham E Wunderink R Silverman H Perl TM Nasraway S Levy H Bone R Wenzel RP Balk R Allred R Pennington JE Wherry JC Bellamy P Cryer H Busuttil R Winston D Perry C Leeper KV Jones C Martin M Tuma P Baird I Brooks J Baird R Rangel S Wagner N Costigan M Gutierrez G Johnson P Efficacy and safety of monoclonal antibody to human tumor necrosis factor alpha in patients with sepsis syndrome. A randomized, controlled, double-blind, multicenter clinical trial. TNF-alpha MAb sepsis study group JAMA 1995 273 934 941 10.1001/jama.1995.03520360048038 7884952

[B12] Reinhart K Wiegand-Löhnert C Grimminger F Kaul M Withington S Treacher D Eckart J Willatts S Bouza C Krausch D Stockenhuber F Eiselstein J Daum L Kempeni J Assessment of the safety and efficacy of the monoclonal anti-tumor necrosis factor antibody-fragment, MAK 195 F, in patients with sepsis and septic shock: a multicenter, randomized, placebo-controlled, dose-ranging study Crit Care Med 1996 24 733 742 10.1097/00003246-199605000-00003 8706447

[B13] Tetta C Gianotti L Cavaillon JM Wratten ML Fini M Braga M Bisagni P Giavaresi G Bolzani R Giardino R Continuous plasmafiltration coupled with sorbent adsorption in a rabbit model of endotoxic shock Crit Care Med 2000 28 1526 1533 10.1097/00003246-200005000-00045 10834707

[B14] Lorente JA Marshall JC Neutralization of tumor necrosis factor in preclinical models of sepsis Shock 2005 24 107 119 10.1097/01.shk.0000191343.21228.78 16374382

[B15] Miles RH Paxton TP Dries DJ Gamelli RL Interferon-gamma increases mortality following cecal ligation and puncture J Trauma 1994 36 607 611 10.1097/00005373-199405000-00001 8189458

[B16] Spight D Trapnell B Zhao B Berclaz P Shanley TP Granulocyte-macrophage-colony-stimulating factor-dependent peritoneal macrophage responses determine survival in experimentally induced peritonitis and sepsis in mice Shock 2008 30 434 442 10.1097/SHK.0b013e3181673543 18277945 PMC2743401

[B17] Reim D Westenfelder K Kaiser-Moore S Schlautkotter S Holzmann B Weighardt H Role of T cells for cytokine production and outcome in a model of acute septic peritonitis Shock 2009 31 245 250 10.1097/SHK.0b013e31817fd02c 18650777

[B18] van Schaik SM Abbas AK Role of T cells in a murine model of Escherichia coli sepsis Eur J Immunol 2007 37 3101 3110 10.1002/eji.200737295 17948264

[B19] Heuer JG Zhang T Zhao J Ding C Cramer M Justen KL Vonderfecht SL Na S Adoptive transfer of in vitro-stimulated CD4 + CD25+ regulatory T cells increases bacterial clearance and improves survival in polymicrobial sepsis J Immunol 2005 174 7141 7146 15905557 10.4049/jimmunol.174.11.7141

[B20] Hiraki S Ono S Tsujimoto H Kinoshita M Takahata R Miyazaki H Saitoh D Hase K Neutralization of interleukin-10 or transforming growth factor-beta decreases the percentages of CD4+ CD25+ Foxp3+ regulatory T cells in septic mice, thereby leading to an improved survival Surgery 2012 151 313 322 10.1016/j.surg.2011.07.019 21982068

[B21] Souza-Fonseca-Guimaraes F Adib-Conquy M Cavaillon JM Natural killer (NK) cells in antibacterial innate immunity: angels or devils? Mol Med 2012 18 270 285 22105606 10.2119/molmed.2011.00201 PMC3324953

[B22] Scumpia PO McAuliffe PF O’Malley KA Ungaro R Uchida T Matsumoto T Remick DG Clare-Salzler MJ Moldawer LL Efron PA CD11c + dendritic cells are required for survival in murine polymicrobial sepsis J Immunol 2005 175 3282 3286 16116220 10.4049/jimmunol.175.5.3282

[B23] Maier M Wutzler S Bauer M Trendafilov P Henrich D Marzi I Altered gene expression patterns in dendritic cells after severe trauma: implications for systemic inflammation and organ injury Shock 2008 30 344 351 10.1097/SHK.0b013e3181673eb4 18323745

[B24] Hotchkiss RS Swanson PE Knudson CM Chang KC Cobb JP Osborne DF Zollner KM Buchman TG Korsmeyer SJ Karl IE Overexpression of Bcl-2 in transgenic mice decreases apoptosis and improves survival in sepsis J Immunol 1999 162 4148 4156 10201940

[B25] Chen W Frank M Jin W Wahl S TGF-beta released by apoptotic T cells contributes to an immunosuppressive milieu Immunity 2001 14 715 725 10.1016/S1074-7613(01)00147-9 11420042

[B26] Ren Y Xie Y Jiang G Fan J Yeung J Li W Tam PK Savill J Apoptotic cells protect mice against lipopolysaccharide-induced shock J Immunol 2008 180 4978 4985 18354223 10.4049/jimmunol.180.7.4978

[B27] Byrne A Reen DJ Lipopolysaccharide induces rapid production of IL-10 by monocytes in the presence of apoptotic neutrophils J Immunol 2002 168 1968 1977 11823533 10.4049/jimmunol.168.4.1968

[B28] Perl M Chung CS Lomas-Neira J Rachel TM Biffl WL Cioffi WG Ayala A Silencing of Fas, but not caspase-8, in lung epithelial cells ameliorates pulmonary apoptosis, inflammation, and neutrophil influx after hemorrhagic shock and sepsis Am J Pathol 2005 167 1545 1559 10.1016/S0002-9440(10)61240-0 16314469 PMC1613198

[B29] Glynne PA Evans TJ Inflammatory cytokines induce apoptotic and necrotic cell shedding from human proximal tubular epithelial cell monolayers Kidney Int 1999 55 2573 2597 10.1046/j.1523-1755.1999.00456.x 10354308

[B30] Husain KD Stromberg PE Woolsey CA Turnbull IR Dunne WM Javadi P Buchman TG Karl IE Hotchkiss RS Coopersmith CM Mechanisms of decreased intestinal epithelial proliferation and increased apoptosis in murine acute lung injury Crit Care Med 2005 33 2350 2357 10.1097/01.CCM.0000182797.89252.A3 16215392 PMC1317567

[B31] Buerke U Carter JM Schlitt A Russ M Schmidt H Sibelius U Grandel U Grimminger F Seeger W Mueller-Werdan U Werdan K Buerke M Apoptosis contributes to septic cardiomyopathy and is improved by simvastatin therapy Shock 2008 29 497 503 10.1097/SHK.0b013e318142c434 18598004

[B32] Zhou M Simms HH Wang P Adrenomedullin and adrenomedullin binding protein-1 attenuate vascular endothelial cell apoptosis in sepsis Ann Surg 2004 240 321 330 10.1097/01.sla.0000133253.45591.5b 15273558 PMC1356410

[B33] Sharshar T Gray F Lorin De La Grandmaison G Hopkinson NS Ross E Dorandeu A Orlikowski D Raphael JC Gajdos P Annane D Apoptosis of neurons in cardiovascular autonomic centres triggered by inducible nitric oxide synthase after death from septic shock Lancet 2003 362 1799 1805 10.1016/S0140-6736(03)14899-4 14654318

[B34] Benjamim CF Canetti C Cunha FQ Kunkel SL Peters-Golden M Opposing and hierarchical roles of leukotrienes in local innate immune versus vascular responses in a model of sepsis J Immunol 2005 174 1616 1620 15661924 10.4049/jimmunol.174.3.1616

[B35] Park SW Chen SW Kim M Brown KM Kolls JK D’Agati VD Lee HT Cytokines induce small intestine and liver injury after renal ischemia or nephrectomy Lab Invest 2011 91 63 84 10.1038/labinvest.2010.151 20697374 PMC2991383

[B36] Lee HT Kim M Kim JY Brown KM Ham A D’Agati VD Mori-Akiyama Y Critical role of interleukin-17A in murine intestinal ischemia-reperfusion injury Am J Physiol Gastrointest Liver Physiol 2013 304 G12 G25 10.1152/ajpgi.00201.2012 23125155

[B37] Bone RC Grodzin CJ Balk RA Sepsis: a new hypothesis for pathogenesis of the disease process Chest 1997 121 235 243 9228382 10.1378/chest.112.1.235

[B38] Cavaillon J-M Adib-Conquy M Cloëz-Tayarani I Fitting C Immunodepression in sepsis and SIRS assessed by ex vivo cytokine production is not a generalized phenomenon: a review J Endotoxin Res 2001 7 85 93 11521088

[B39] Tamayo E Fernández A Almansa R Carrasco E Heredia M Lajo C Goncalves L Gómez-Herreras JI de Lejarazu RO Bermejo-Martin JF Pro- and anti-inflammatory responses are regulated simultaneously from the first moments of septic shock Eur Cytokine Netw 2011 22 82 87 21628135 10.1684/ecn.2011.0281

[B40] Tang BM Huang SJ McLean AS Genome-wide transcription profiling of human sepsis: a systematic review Crit Care 2010 14 R237 10.1186/cc9392 21190579 PMC3219990

[B41] Muñoz C Carlet J Fitting C Misset B Bleriot JP Cavaillon JM Dysregulation of in vitro cytokine production by monocytes during sepsis J Clin Invest 1991 88 1747 1754 10.1172/JCI115493 1939659 PMC295719

[B42] Kim OY Monsel A Bertrand M Coriat P Cavaillon JM Adib-Conquy M Differential down-regulation of HLA-DR on monocyte subpopulations during systemic inflammation Crit Care 2010 14 R61 10.1186/cc8959 20385017 PMC2887183

[B43] Kox M Timmermans K Veaneker M Scheffer G Pickkers P Immune paralysis in trauma patients, implications for pre-hospital intervention Crit Care 2013 17 9 10.1186/cc11933

[B44] Adrie C Adib-Conquy M Laurent I Monchi M Vinsonneau C Fitting C Fraisse F Dinh-Xuan AT Carli P Spaulding C Dhainaut JF Cavaillon JM Successful cardiopulmonary resuscitation after cardiac arrest as a ‘sepsis like’ syndrome Circulation 2002 106 562 568 10.1161/01.CIR.0000023891.80661.AD 12147537

[B45] Gentile LF Cuenca AG Efron PA Ang D Bihorac A McKinley BA Moldawer LL Moore FA Persistent inflammation and immunosuppression: a common syndrome and new horizon for surgical intensive care J Trauma Acute Care Surg 2012 72 1491 1501 10.1097/TA.0b013e318256e000 22695412 PMC3705923

[B46] Danikas DD Karakantza M Theodorou GL Sakellaropoulos GC Gogos CA Prognostic value of phagocytic activity of neutrophils and monocytes in sepsis. Correlation to CD64 and CD14 antigen expression Clin Exp Immunol 2008 154 87 97 10.1111/j.1365-2249.2008.03737.x 18727624 PMC2561092

[B47] Adib-Conquy M Adrie C Fitting C Gattoliat O Beyaert R Cavaillon JM Up-regulation of MyD88s and SIGIRR, molecules inhibiting Toll-like receptor signaling, in monocytes from septic patients Crit Care Med 2006 34 2377 2385 10.1097/01.CCM.0000233875.93866.88 16850005

[B48] Peck OM Fan H Tempel GE Teti G Halushka PV Cook JA Staphylococcus aureus and lipopolysaccharide induce homologous tolerance but heterologous priming: role of interferon-gamma Shock 2004 21 254 260 10.1097/01.shk.0000111662.09279.59 14770039

[B49] Quintin J Saeed S Martens JH Giamarellos-Bourboulis EJ Ifrim DC Logie C Jacobs L Jansen T Kullberg BJ Wijmenga C Joosten LA Xavier RJ van der Meer JW Stunnenberg HG Netea MG Candida albicans infection affords protection against reinfection via functional reprogramming of monocytes Cell Host Microbe 2012 12 223 232 10.1016/j.chom.2012.06.006 22901542 PMC3864037

[B50] Cavaillon JM Adrie C Fitting C Adib-Conquy M Reprogramming of circulatory cells in sepsis and SIRS J Endotoxin Res 2005 11 311 320 10.1177/09680519050110050901 16263005

[B51] Suzuki T Shimizu T Szalay L Choudhry MA Rue LW 3rd Bland KI Chaudry IH Androstenediol ameliorates alterations in immune cells cytokine production capacity in a two-hit model of trauma-hemorrhage and sepsis Cytokine 2006 34 76 84 10.1016/j.cyto.2006.04.007 16737821

[B52] Neunaber C Oestern S Andruszkow H Zeckey C Mommsen P Kutter D Stöfen M Krettek C Hildebrand F Cytokine productive capacity of alveolar macrophages and Kupffer cells after femoral fracture and blunt chest trauma in a murine trauma model Immunol Lett 2013 152 159 166 10.1016/j.imlet.2013.05.012 23735227

[B53] Philippart F Fitting C Cavaillon JM Lung microenvironment contributes to the resistance of alveolar macrophages to develop tolerance to endotoxin* Crit Care Med 2012 40 2987 2996 10.1097/CCM.0b013e31825b8d57 22878679

[B54] Hoogerwerf JJ de Vos AF van’t Veer C Bresser P de Boer A Tanck MW Draing C van der Zee JS van der Poll T Priming of alveolar macrophages upon instillation of lipopolysaccharide in the human lung Am J Respir Cell Mol Biol 2010 42 349 356 10.1165/rcmb.2008-0362OC 19448156

[B55] Zager RA Johnson AC Lund S ‘Endotoxin tolerance’: TNF-alpha hyper-reactivity and tubular cytoresistance in a renal cholesterol loading state Kidney Int 2007 71 496 503 10.1038/sj.ki.5002092 17228359

[B56] Zurovsky Y Eligal Z Reduction of risk following endotoxin injection in unilaterally nephrectomized rats Exp Toxicol Pathol 1996 48 41 46 10.1016/S0940-2993(96)80092-3 8919270

[B57] Zhang S Kim CC Batra S McKerrow JH Loke P Delineation of diverse macrophage activation programs in response to intracellular parasites and cytokines PLoS Negl Trop Dis 2010 4 e648 10.1371/journal.pntd.0000648 20361029 PMC2846935

[B58] Hacham M Cristal N White RM Segal S Apte RN Complementary organ expression of IL-1 vs IL-6 and CSF-1 activities in normal and LPS injected mice Cytokine 1996 8 21 31 10.1006/cyto.1995.0004 8742063

[B59] Raschke RA Garcia-Orr R Hemophagocytic lymphohistiocytosis: a potentially underrecognized association with systemic inflammatory response syndrome, severe sepsis, and septic shock in adults Chest 2011 140 933 938 10.1378/chest.11-0619 21737492

[B60] Ding Y Chung CS Newton S Chen Y Carlton S Albina JE Ayala A Polymicrobial sepsis induces divergent effects on splenic and peritoneal dendritic cell function in mice Shock 2004 22 137 144 10.1097/01.shk.0000131194.80038.3f 15257086 PMC2253681

[B61] Metzger DW Salmon SL Kirimanjeswara G Differing effects of interleukin-10 on cutaneous and pulmonary Francisella tularensis live vaccine strain infection Infect Immun 2013 81 2022 2027 10.1128/IAI.00024-13 23529615 PMC3676042

[B62] Blanchet C Jouvion G Fitting C Cavaillon JM Adib-Conquy M Protective or deleterious role of scavenger receptors SR-A and CD36 on host resistance to *Staphylococcus aureus* depends on the site of infection PloS One 2014 9 e87927 10.1371/journal.pone.0087927 24498223 PMC3909292

[B63] Dorrington MG Roche AM Chauvin SE Tu Z Mossman KL Weiser JN Bowdish DM MARCO is required for TLR2- and Nod2-mediated responses to *Streptococcus pneumoniae* and clearance of pneumococcal colonization in the murine nasopharynx J Immunol 2013 190 250 258 10.4049/jimmunol.1202113 23197261 PMC3529821

[B64] Pang G Ortega M Zighang R Reeves G Clancy R Autocrine modulation of IL-8 production by sputum neutrophils in chronic bronchial sepsis Am J Respir Crit Care Med 1997 155 726 371 10.1164/ajrccm.155.2.9032219 9032219

[B65] Muenzer JT Davis CG Chang K Schmidt RE Dunne WM Coopersmith CM Hotchkiss RS Characterization and modulation of the immunosuppressive phase of sepsis Infect Immun 2010 78 1582 15492 10.1128/IAI.01213-09 20100863 PMC2849407

[B66] Warren HS Fitting C Hoff E Adib-Conquy M Beasley-Topliffe L Tesini B Liang X Valentine C Hellman J Hayden D Cavaillon JM Resilience to bacterial infection: difference between species could be due to proteins in serum J Infect Dis 2010 201 223 232 10.1086/649557 20001600 PMC2798011

[B67] Seok J Warren HS Cuenca AG Mindrinos MN Baker HV Xu W Richards DR McDonald-Smith GP Gao H Hennessy L Finnerty CC López CM Honari S Moore EE Minei JP Cuschieri J Bankey PE Johnson JL Sperry J Nathens AB Billiar TR West MA Jeschke MG Klein MB Gamelli RL Gibran NS Brownstein BH Miller-Graziano C Calvano SE Mason PH Genomic responses in mouse models poorly mimic human inflammatory diseases Proc Natl Acad Sci U S A 2013 110 3507 3512 10.1073/pnas.1222878110 23401516 PMC3587220

[B68] Kapetanovic R Fairbairn L Beraldi D Sester DP Archibald AL Tuggle CK Hume DA Pig bone marrow-derived macrophages resemble human macrophages in their response to bacterial lipopolysaccharide J Immunol 2012 188 3382 3394 10.4049/jimmunol.1102649 22393154

[B69] Takahashi H Tsuda Y Takeuchi D Kobayashi M Herndon DN Suzuki F Influence of systemic inflammatory response syndrome on host resistance against bacterial infections Crit Care Med 2004 32 1879 1885 10.1097/01.CCM.0000139606.34631.61 15343016

[B70] Otto GP Sossdorf M Claus RA Rödel J Menge K Reinhart K Bauer M Riedemann NC The late phase of sepsis is characterized by an increased microbiological burden and death rate Crit Care 2011 15 R183 10.1186/cc10332 21798063 PMC3387626

[B71] Osawa R Singh N Cytomegalovirus infection in critically ill patients: a systematic review Crit Care 2009 13 R68 10.1186/cc7875 19442306 PMC2717427

[B72] Luyt CE Combes A Deback C Aubriot-Lorton MH Nieszkowska A Trouillet JL Capron F Agut H Gibert C Chastre J Herpes simplex virus lung infection in patients undergoing prolonged mechanical ventilation Am J Respir Crit Care Med 2007 175 935 942 10.1164/rccm.200609-1322OC 17234903

[B73] Torgersen C Moser P Luckner G Mayr V Jochberger S Hasibeder WR Dünser MW Macroscopic postmortem findings in 235 surgical intensive care patients with sepsis Anesth Analg 2009 108 1841 1847 10.1213/ane.0b013e318195e11d 19448210

[B74] Döcke WD Randow F Syrbe U Krausch D Asadullah K Reinke P Volk HD Kox W Monocyte deactivation in septic patients: restoration by IFNg treatment Nature Med 1997 3 678 681 10.1038/nm0697-678 9176497

[B75] Wasserman D Ioannovich JD Hinzmann RD Deichsel G Steinmann GG Interferon-gamma in the prevention of severe burn-related infections: a European phase III multicenter trial. The Severe Burns Study Group Crit Care Med 1998 26 434 439 10.1097/00003246-199803000-00010 9504568

[B76] Bo L Wang F Zhu J Li J Deng X Granulocyte-colony stimulating factor (G-CSF) and granulocyte-macrophage colony stimulating factor (GM-CSF) for sepsis: a meta-analysis Crit Care 2011 15 R58 10.1186/cc10031 21310070 PMC3221991

[B77] Verhoef G Boogaerts M Treatment with granulocyte-macrophage colony stimulating factor and the adult respiratory distress syndrome Am J Hematol 1991 36 285 287 10.1002/ajh.2830360413 1672790

[B78] Bozinovski S Jones J Vlahos R Hamilton J Anderson G Granulocyte/macrophage-colony-stimulating factor (GM-CSF) regulates lung innate immunity to lipopolysaccharide through Akt/Erk activation of NFkappa B and AP-1 in vivo J Biol Chem 2002 277 42808 42814 10.1074/jbc.M207840200 12208854

[B79] Khameneh HJ Isa SA Min L Nih FW Ruedl C GM-CSF signalling boosts dramatically IL-1 production PLoS One 2011 6 e23025 10.1371/journal.pone.0023025 21829580 PMC3145786

[B80] Venet F Foray AP Villars-Méchin A Malcus C Poitevin-Later F Lepape A Monneret G IL-7 restores lymphocyte functions in septic patients J Immunol 2012 189 5073 5081 10.4049/jimmunol.1202062 23053510

[B81] Sinha ML Fry TJ Fowler DH Miller G Mackall CL Interleukin 7 worsens graft-versus-host disease Blood 2002 100 2642 2649 10.1182/blood-2002-04-1082 12239180

[B82] Willis CR Seamons A Maxwell J Treuting PM Nelson L Chen G Phelps S Smith CL Brabb T Iritani BM Maggio-Price L Interleukin-7 receptor blockade suppresses adaptive and innate inflammatory responses in experimental colitis J Inflamm (Lond) 2012 9 39 10.1186/1476-9255-9-39 23057802 PMC3551718

[B83] Hartgring SA Willis CR Alcorn D Nelson LJ Bijlsma JW Lafeber FP van Roon JA Blockade of the interleukin-7 receptor inhibits collagen-induced arthritis and is associated with reduction of T cell activity and proinflammatory mediators Arthritis Rheum 2010 62 2716 2725 10.1002/art.27578 20499386

[B84] Jin JO Yu Q Systemic administration of TLR3 agonist induces IL-7 expression and IL-7-dependent CXCR3 ligand production in the lung J Leukoc Biol 2013 93 413 425 10.1189/jlb.0712360 23271706 PMC4050520

[B85] Alderson MR Tough TW Ziegler SF Grabstein KH Interleukin 7 induces cytokine secretion and tumoricidal activity by human peripheral blood monocytes J Exp Med 1991 173 923 930 10.1084/jem.173.4.923 2007858 PMC2190815

[B86] Ostatin A Paltsev A Leplina O Shevela Y Chernykh H The experience of surgical infections treatment with extracorporal immunotherapy Medicinskaya Immunol 2000 2 43 51

[B87] Ertel W Morrison MH Ayala A Chaudry IH Chloroquine attenuates hemorrhagic shock-induced immunosuppression and decreases susceptibility to sepsis Arch Surg 1992 127 70 75 10.1001/archsurg.1992.01420010084012 1734852

[B88] Suzuki T Shimizu T Yu HP Hsieh YC Choudhry MA Schwacha MG Chaudry IH Tissue compartment-specific role of estrogen receptor subtypes in immune cell cytokine production following trauma-hemorrhage J Appl Physiol 2007 102 163 168 17023568 10.1152/japplphysiol.00964.2006

[B89] Chiang N Fredman G Bäckhed F Oh SF Vickery T Schmidt BA Serhan CN Infection regulates pro-resolving mediators that lower antibiotic requirements Nature 2012 484 524 528 10.1038/nature11042 22538616 PMC3340015

[B90] Eisen DP Reid D McBryde ES Acetyl salicylic acid usage and mortality in critically ill patients with the systemic inflammatory response syndrome and sepsis Crit Care Med 2012 40 1761 1767 10.1097/CCM.0b013e318246b9df 22610182

[B91] Wu J Zhou L Liu J Ma G Kou Q He Z Chen J Ou-Yang B Chen M Li Y Wu X Gu B Chen L Zou Z Qiang X Chen Y Lin A Zhang G Guan X The efficacy of thymosin alpha 1 for severe sepsis (ETASS): a multicenter, single-blind, randomized and controlled trial Crit Care 2013 17 R8 10.1186/cc11932 23327199 PMC4056079

[B92] Zhang Y Chen H Li YM Zheng SS Chen YG Li LJ Zhou L Xie HY Praseedom RK Thymosin alpha1- and ulinastatin-based immunomodulatory strategy for sepsis arising from intra-abdominal infection due to carbapenem-resistant bacteria J Infect Dis 2008 198 723 730 10.1086/590500 18613793

[B93] Sodhi A Paul S Involvement of mitogen-activated protein kinases in the signal transduction pathway of bone marrow-derived macrophage activation in response to in vitro treatment with thymosin alpha 1 Int Immunopharmacol 2002 2 47 58 10.1016/S1567-5769(01)00139-4 11789669

[B94] Yang X Qian F He HY Liu KJ Lan YZ Ni B Tian Y Fu XL Zhang J Shen ZG Li J Yin Y Li JT Wu YZ Effect of thymosin alpha-1 on subpopulations of Th1, Th2, Th17, and regulatory T cells (Tregs) in vitro Braz J Med Biol Res 2012 45 25 32 10.1590/S0100-879X2011007500159 22245858 PMC3854146

[B95] Monaghan SF Thakkar RK Tran ML Huang X Cioffi WG Ayala A Heffernan DS Programmed death 1 expression as a marker for immune and physiological dysfunction in the critically ill surgical patient Shock 2012 38 117 122 10.1097/SHK.0b013e31825de6a3 22683728

[B96] Zhang Y Li J Lou J Zhou Y Bo L Zhu J Zhu K Wan X Cai Z Deng X Upregulation of programmed death-1 on T cells and programmed death ligand-1 on monocytes in septic shock patients Crit Care 2011 15 R70 10.1186/cc10059 21349174 PMC3222003

[B97] Guignant C Lepape A Huang X Kherouf H Denis L Poitevin F Malcus C Chéron A Allaouchiche B Gueyffier F Ayala A Monneret G Venet F Programmed death-1 levels correlate with increased mortality, nosocomial infection and immune dysfunctions in septic shock patients Crit Care 2011 15 R99 10.1186/cc10112 21418617 PMC3219369

[B98] Huang X Venet F Wang YL Lepape A Yuan Z Chen Y Swan R Kherouf H Monneret G Chung CS Ayala A PD-1 expression by macrophages plays a pathologic role in altering microbial clearance and the innate inflammatory response to sepsis Proc Natl Acad Sci U S A 2009 106 6303 6308 10.1073/pnas.0809422106 19332785 PMC2669369

[B99] Brahmamdam P Inoue S Unsinger J Chang KC McDunn JE Hotchkiss RS Delayed administration of anti-PD-1 antibody reverses immune dysfunction and improves survival during sepsis J Leukoc Biol 2010 88 233 240 10.1189/jlb.0110037 20483923 PMC6607999

[B100] Zhang Y Zhou Y Lou J Li J Bo L Zhu K Wan X Deng X Cai Z PD-L1 blockade improves survival in experimental sepsis by inhibiting lymphocyte apoptosis and reversing monocyte dysfunction Crit Care 2010 14 R220 10.1186/cc9354 21118528 PMC3220038

[B101] Zhu W Bao R Fan X Tao T Zhu J Wang J Li J Bo L Deng X PD-L1 blockade attenuated sepsis-induced liver injury in a mouse cecal ligation and puncture model Mediators Inflamm 2013 2013 361501 24324295 10.1155/2013/361501 PMC3844221

[B102] Manjuck J Saha DC Astiz M Eales LJ Rackow EC Decreased response to recall antigens is associated with depressed costimulatory receptor expression in septic critically ill patients J Lab Clin Med 2000 135 153 160 10.1067/mlc.2000.104306 10695660

[B103] Inoue S Bo L Bian J Unsinger J Chang K Hotchkiss RS Dose-dependent effect of anti-CTLA-4 on survival in sepsis Shock 2011 36 38 44 10.1097/SHK.0b013e3182168cce 21368717 PMC3412306

[B104] Chang KC Burnham CA Compton SM Rasche DP Mazuski R Smcdonough J Unsinger J Korman AJ Green JM Hotchkiss RS Blockade of the negative co-stimulatory molecules PD-1 and CTLA-4 improves survival in primary and secondary fungal sepsis Crit Care 2013 17 R85 10.1186/cc12711 23663657 PMC3706819

[B105] LeMessurier K Häcker H Tuomanen E Redecke V Inhibition of T cells provides protection against early invasive pneumococcal disease Infect Immun 2010 78 5287 5294 10.1128/IAI.00431-10 20855509 PMC2981332

[B106] Shubin NJ Monaghan SF Heffernan DS Chung CS Ayala A B and T lymphocyte attenuator expression on CD4+ T-cells associates with sepsis and subsequent infections in ICU patients Crit Care 2013 17 R276 10.1186/cc13131 24289156 PMC4057112

[B107] Shubin NJ Chung CS Heffernan DS Irwin LR Monaghan SF Ayala A BTLA expression contributes to septic morbidity and mortality by inducing innate inflammatory cell dysfunction J Leukoc Biol 2012 92 593 603 10.1189/jlb.1211641 22459947 PMC3427605

[B108] Kobayashi Y Iwata A Suzuki K Suto A Kawashima S Saito Y Owada T Kobayashi M Watanabe N Nakajima H B and T lymphocyte attenuator inhibits LPS-induced endotoxic shock by suppressing Toll-like receptor 4 signaling in innate immune cells Proc Natl Acad Sci U S A 2013 110 5121 5126 10.1073/pnas.1222093110 23479601 PMC3612598

